# Paraclinoid aneurysm clipping: how I do it

**DOI:** 10.1007/s00701-025-06609-1

**Published:** 2025-07-21

**Authors:** Bruno Vernile, Kyle McGrath, Sudhakar Vadivelu, Mario Zuccarello

**Affiliations:** 1 Division of Pediatric Neurosurgery, Department of Neurosurgery, Cincinnati Children’s Hospital Medical Center, University of Cincinnati College of Medicine, Cincinnati, OH USA; 2 Department of Neurosurgery, University of Cincinnati, Ohio, OH USA

**Keywords:** Paraclinoid aneurysm, Aneurysm clipping, Cerebral aneurysm, Surgical approach

## Abstract

Paraclinoid aneurysms continue to present a surgical challenge, even in the modern endovascular era. Although these aneurysms are uncommon, surgical clipping remains the treatment of choice in select cases. Successful surgical management requires both extensive experience and a comprehensive understanding of paraclinoid anatomy. In this report, we outline our approach to planning and surgical techniques for managing paraclinoid aneurysms.

**Supplementary Information:**

The online version contains supplementary material available at 10.1007/s00701-025-06609-1.

## Introduction

Paraclinoid aneurysms are internal carotid artery (ICA) aneurysms that develop near the anterior clinoid process (ACP), encompassing the ICA’s clinoid and ophthalmic segments. While rare, managing these aneurysms surgically is challenging due to the dense network of vascular and neural structures encased by intricate bony prominences in the paraclinoid region. This complexity contributes to a heightened risk of morbidity and mortality. Although modern endovascular techniques have facilitated treatment, surgical clipping remains the preferred approach in select cases. An in-depth understanding of the paraclinoid region’s anatomy, combined with surgical expertise, is crucial for successful intervention [1].

This article describes our planning and surgical technique to manage aneurysms in the paraclinoid region.

## Relevant surgical anatomy

### ACP and Nearby Dural Folds

The ACP is a pyramidal-shaped bony prominence with three roots: laterally, it continues with the lesser wing of the sphenoid; anteromedially, it articulates with the body of the sphenoid at the sphenoidal planum, defining the roof of the optic canal (OC); inferomedially, it articulates with the body of the sphenoid, delineating the optic strut (OS), which is a bony segment that divides the optic foramen medially, and the superior orbital fissure (SOF) laterally.

Dural folds:

- *Proximal dural ring* (PDR) or *clinoid-oculomotor membrane* (COM), from ACP’s periosteum to the ICA and the oculomotor nerve.
- *Distal dural ring* (DDR), dura mater from ACP that encircles ICA’s adventitia. It defines the transition between the extradural and intradural ICA.
- *Falciform ligament*, dura mater from ACP to digraph sellae, and sphenoidal planum.
- *Interclinoid ligament* and the *anterior petroclinoid ligament*, respectively from the insertion of the medial margin of the tentorium between the anterior and posterior clinoid processes and the apex of the petrous bone and ACP.

### ICA ophthalmic segment

The ophthalmic segment extends from DDR to the origin of the posterior communicating artery (PCoA). The first major branch that arises from this arterial segment is the *ophthalmic artery* (OphA). Rarely, the OphA may originate from the clinoid segment of the ICA, the middle meningeal artery, or the cavernous segment of the ICA. Regardless of where the OphA originates, it enters the orbit through the optic canal [5].

An average of four perforating branches arises from the posterior and medial walls of ICA, directed toward the pituitary stalk, optic chiasm, and the pre-mammillary region of the third ventricle, as well as the optic nerve. Perforators directed toward the infundibulum of the pituitary gland are referred to as the superior hypophyseal arteries (typically 1–2 branches). The major perforator, the *superior hypophyseal artery* (SHA), supplies the infundibulum and the anterior portion of the pituitary gland [4].

### ICA clinoid segment

It extends from COM through DDR. It is covered by a venous clinoid plexus, related to the cavernous sinus; rarely it gives rise to the OphtA [6].

### Description of the technique

#### Preoperative assessment

A *CT angiography* provides information on:

- the aneurysm’s surrounding bony anatomy.
- the presence of calcified dural folds or aneurysm wall.
- ACP pneumatization.
- aneurysm relationship with skull base and OS, to define if there is an intradural component.

The *Digital Subtraction Angiography* (DSA) provides more detailed features of the aneurysm and further allows:

- to perform a *balloon occlusion test* (BOT).
- to evaluate cervical ICA segment for proximal control (height of carotid bifurcation, carotid stenosis).

The MRI is reserved for unruptured aneurysms to evaluate:

- aneurysms relationships with surrounding neuronal structures.
- real size in thrombosed aneurysms.

#### Balloon Occlusion Test

A BOT is used to assess whether compensatory blood flow can adequately supply the brain if ICA is blocked [7]. During the test, a small balloon catheter is temporarily inflated within the ICA presenting the aneurysm, stopping blood flow while monitoring clinical and radiographic response, and i.v. 99-TcHMPAO is injected. This determines if the patient can tolerate permanent occlusion of the ICA. During the test, the patient is examined clinically, and collateral flow is assessed via angiography. After the test, a single photon emission computed tomography (SPECT) scan is done to assess symmetry of cerebral circulation.

#### Pre-procedure planning

- Use IONM to monitor neurological function.
- Position the patient supine, turning the head no more than 15 degrees toward the approach’s contralateral side.
- Place the head in slight extension, as excessive extension of the head can restrict visualization of the ACP.
- Include the neck in the surgical field to prepare the *external carotid artery* (ECA) and the *common carotid artery* (CCA) in case of ligation to make the aneurysm’s dome softer or unexpected intraoperative rupture.
- Isolate and protect the *superior temporal artery* (STA) on exposure, in case a bypass is needed.

#### Craniotomy

A pterional approach allows to access the paraclinoid region.

#### Clinoidectomy

**Fig. 1:**
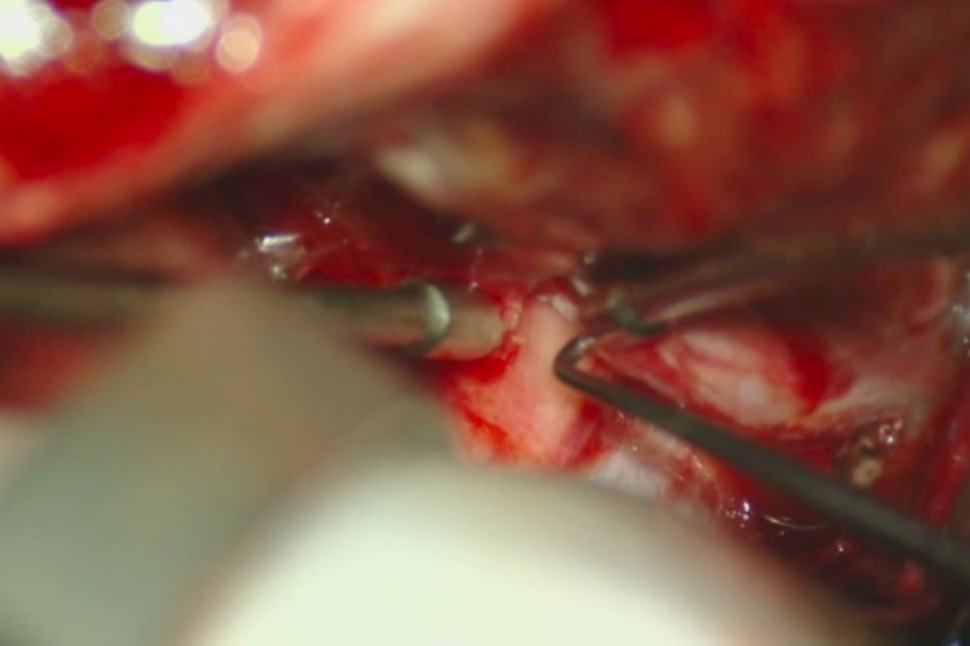
Release the optic nerve by drilling the anterior clinoid process and opening the falciform ligament

An anterior clinoidectomy reduces the risk of the ON injury and it is tailored to the size and location of the aneurysm [2, 3]; it could be completed via an intradural or an extradural route depending on the aneurysm’s presentation and its dome’s projection (Fig. 1). Post-clipping, muscle placement over the clinoid tip minimizes rhinorrhea risk.

- ***Intradural clinoidectomy:*** in cases of ruptured aneurysms where brain swelling can impede access to the extradural clinoid. It allows the surgeon to gain continuous control of the aneurysm during the clinoidectomy.
- ***Extradural clinoidectomy:*** provides wide maneuverability for the following intradural aneurysm dissection thus reducing the surgical stress on the brain parenchyma. Together with CSF diversion, it relaxes the brain and provides a wider exposure to the paraclinoid region [8]***.***

#### Type and clip selection

**Fig. 2:**
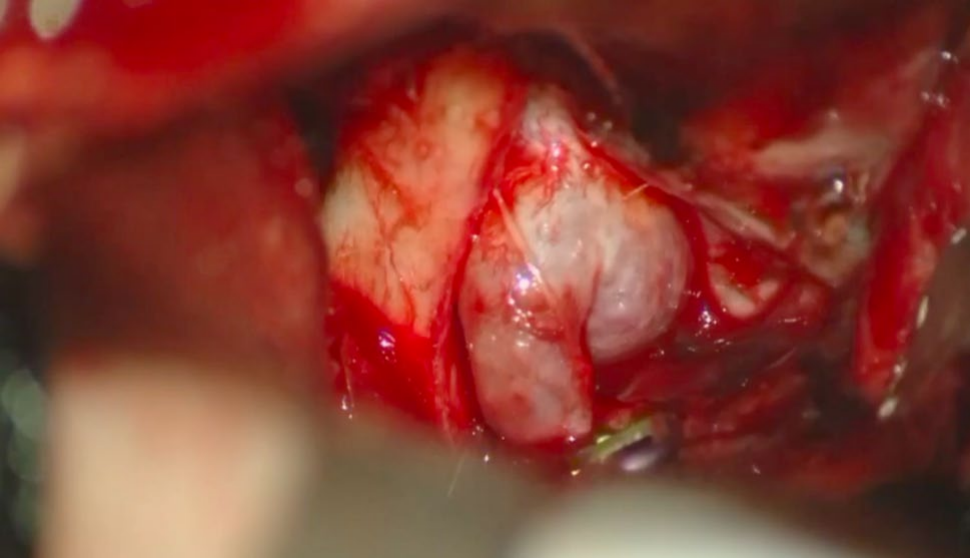
After the cervical carotid is clamped at the neck, a temporary clip is applied distal to the aneurysm

**Fig. 3:**
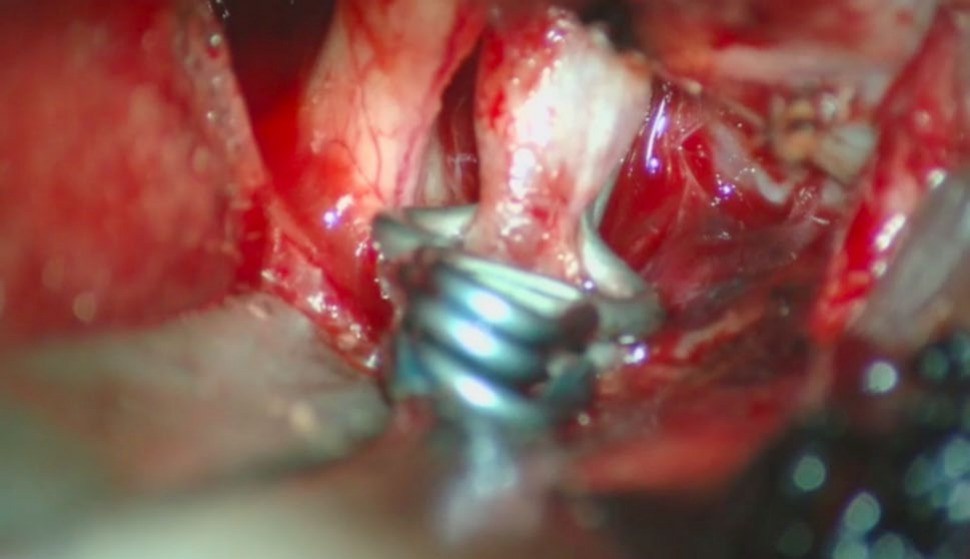
Clipping of the aneurysm with right-angled clips
- *Lateral projecting aneurysms or transitional aneurysms* (also known as *subclinoid aneurysms*): it is safer for the patient an endovascular approach because of their relationship with the cavernous sinus.

- *Dorsal/Superior projecting aneurysms*: intradural clinoidectomy may be safer because of the geometric relationship between the aneurysm’s dome and the inferior aspect of the ACP. A more lateral approach to the aneurysm rather than the typical anterolateral trans-Sylvian approach is recommended. A gentle-curved clip parallel to the ICA is suggested.
- *Ventral/Inferior projecting aneurysms*: fenestrated clip placement may be beneficial, paying close attention to the possibility of adherent PComA or SHA perforators out of direct visualization to the surgeon.
- *Medial projecting aneurysms*: a right-angled clip allows to see the tip of the aneurysm on the ventral side of the carotid (Fig. 2, Fig. 3).

#### Post‑operative follow‑up

- Euvolemia, BP control and daily TCDs and DSA in case of evidence of vasospasm for ruptured aneurysms.
- 24 h observation in ICU and post-operative CT scan for unruptured aneurysms.

### Surgical indications

- Ruptured intradural aneurysm (no endovascular indication)
- Optic nerve compression due to aneurysm mass effect
- Young patients
- Multiple intracranial aneurysm
- Intradural unruptured aneurysm with size > 7 mm

### Limitations

- Ventral projecting and transitional aneurysm
- Calcified aneurysm wall
- Elderly (> 65 years old)
- Asymptomatic and small-size aneurysms

### How to avoid complications

1. Include the CCM for proximal control
2. Identify structures that provide complications:
  1. OphtA, PComA, AChoA arteries in cases of giant aneurysms
  2. Dural folds that wrap the ON
3. Use iMDS, FNa-VA, ICG-VA and, in more complex cases, intraoperative angiogram to assess aneurysm occlusion and surrounding aneurysms’ vessels patency.

### Specific information for the patient

Make the patients aware about potential complications:

- Necessity of a by-pass in case of ICA sacrifice
- CSF leakage with the need for further treatment
- Stroke
- Transient/permanent impairment of the eyes’ motility or visual function deterioration
- Skin- and wound-healing complications.

## Key points summary

1. Knowledge of complex regional anatomy (ACP, ON, oculomotor nerve)
2. Knowledge of the complex regional vascular anatomy (OphA, SHA, perforating branches to the ON and chiasm)
3. Understanding of aneurysm and surrounding vessels anatomy on x-rays
4. Evaluation of the contralateral compensation with BTO in case of a pathologic ICA’s sacrifice
5. Proper surgical approach (unruptured vs. ruptured aneurysms, shape, direction)
6. Prepare the STA in case a by-pass is needed
7. Maximal ON decompression (anterior clinoidectomy, release of the DDR and PDR)
8. Proximal ICA control at the neck
9. Assessment of the aneurysm surrounding vessels’ patency after clipping (IONM, FNa-VA, IGC-VA, iMDS, intraoperative angiogram)
10. Assessment of the aneurysm occlusion

## Supplementary Information

Below is the link to the electronic supplementary material.

Supplementary video (MP4 354 mb)

## Data Availability

No datasets were generated or analysed during the current study.

## Abbreviations

*ACP*: Anterior clinoid process
*OC*: Optic canal
*OS*: Optic strut
*SOF*: Superior orbital fissure
*PDR*: Proximal dural ring
*COM*: Carotid-oculomotor membrane
*DDR*: Distal dural ring
*PCoA*: Posterior communicating artery
*AChoA*: Anterior choroidal artery
*OphA*: Ohptalmic artery
*SHA*: Superior hypophysial artery
*BOT*: Balloon occlusion test
*ICA*: Internal carotid artery
*ECA*: External carotid artery
*CCA*: Common carotid artery
*99 m-TcHMPAO*: Technetium 99 m-hexamethylpropyleneamine oxime
*iMDS*: Intraoperative microvascular doppler
*FNa-VA*: Fluorescein sodium videoangiography
*ICG-VA*: Intraoperative indocyanine green videoangiography

## References

[1] Andaluz N, Beretta F, Bernucci C, Keller JT, Zuccarello M (2006) Evidence for the improved exposure of the ophthalmic segment of the internal carotid artery after anterior clinoidectomy: morphometric analysis. Acta Neurochir 148(9):971–5; discussion 975–6. 10.1007/s00701-006-0862-x 10.1007/s00701-006-0862-x 16917665

[2] Batjer HH, Samson DS (1990) Retrograde suction decompression of giant paraclinoidal aneurysms. Technical note. J Neurosurg 73(2):305–306. 10.3171/jns.1990.73.2.0305 10.3171/jns.1990.73.2.0305 2366090

[3] Bhaisora KS, Singh G, Das KK, Srivastava AK (2024) How I do it-Dolenc approach for clipping of ventral wall paraclinoid ICA aneurysm. Acta Neurochir 166(1):406. 10.1007/s00701-024-06297-3 39400784 10.1007/s00701-024-06297-3

[4] Gibo H, Kobayashi S, Kyoshima K, Hokama M (1988) Microsurgical anatomy of the arteries of the pituitary stalk and gland as viewed from above. Acta Neurochir (Wien) 90(1–2):60–66. 10.1007/BF01541268 3344626 10.1007/BF01541268

[5] Renn WH, Rhoton AL Jr (1975) Microsurgical anatomy of the sellar region. J Neurosurg 43(3):288–298. 10.3171/jns.1975.43.3.0288 1151464 10.3171/jns.1975.43.3.0288

[6] Seoane E, Rhoton AL Jr, de Oliveira E (1998) Microsurgical anatomy of the dural collar (carotid collar) and rings around the clinoid segment of the internal carotid artery. Neurosurgery. 42(4):869–84; discussion 884–6. h10.1097/00006123-199804000-00108 10.1097/00006123-199804000-00108 9574652

[7] Skrap M, Petralia B, Toniato G (2010) Temporary balloon occlusion during the surgical treatment of giant paraclinoid and vertebrobasilar aneurysms. *Acta Neurochir (Wien)*. 152(3):435–42. 10.1007/s00701-009-0566-0 10.1007/s00701-009-0566-0 20186525

[8] van Loveren HR, Keller JT, El-Kalliny M, Scodary DJ, Tew JM Jr (1991) The Dolenc technique for cavernous sinus exploration (cadaveric prosection). Technical note. J Neurosurg 74(5):837–844. 10.3171/jns.1991.74.5.0837 2013784 10.3171/jns.1991.74.5.0837

